# Synopiidae Dana, 1853 (Crustacea, Amphipoda) of the Clarion-Clipperton Zone

**DOI:** 10.3897/zookeys.1274.141366

**Published:** 2026-03-24

**Authors:** Lauren Elizabeth Hughes, Anne Helene Solberg Tandberg

**Affiliations:** 1 Invertebrates (non-Insects) section, Natural History Museum, London, Cromwell Road, South Kensington, UK Natural History Museum London United Kingdom; 2 University of Bergen, NO5020 Bergen, Norway University of Bergen Bergen Norway; 3 Senckenberg Research Institute and Nature Museum, Frankfurt, Germany Senckenberg Research Institute and Nature Museum Frankfurt Germany

**Keywords:** *
Austrosyrrhoe
*, deep-sea, depth range, North Eastern Pacific, *
Syrrhoe
*, taxonomy

## Abstract

The Synopiidae*Austrosyrrhoe hamptonae***sp. nov**. and *Syrrhoe manowitzae***sp. nov**. are morphologically described from the Clarion-Clipperton Zone, Pacific Ocean. Their descriptions are supplemented by molecular barcodes. Both *Austrosyrrhoe* and *Syrrhoe* have been recorded from only a few specimens in previous studies. The ten specimens reported here add to the sparse understanding of the Synopiidae. Also remarkable is the depth range of both genera, occurring in shallow waters of ~50 m to depths of ~5000 m. Prior to this study, the known depth range was less than 3000 m for *Austrosyrrhoe* and 3500 m for *Syrrhoe*. In the Clarion-Clipperton Zone the new species *Austrosyrrhoe hamptonae***sp. nov**. was collected at 5109 m and *Syrrhoe manowitzae***sp. nov**. from 4368 m, providing a significant increase in the maximum recorded depths for these genera.

## Introduction

*Austrosyrrhoe* K.H. Barnard, 1925 is a little-known genus of five species with records spanning the breadth of the globe, species reports are only from the original descriptions and total 55 specimens for the genus (Table 1, Fig. 8). *Austrosyrrhoe* has been recorded from depths as shallow as ~58 m in the Irish Sea, and, prior to this study, the deepest record was 2700 m west of Greenland. *Austrosyrrhoe* is here reported from a depth of 5109 m in the Clarion-Clipperton Zone. Two *Austrosyrrhoe* species, *A. crassipes* K.H. Barnard, 1925 and *A. fimbriatus* (Stebbing & Robertson, 1891) have problematic type material (see remarks of *A. hamptonae* sp. nov.) and a limited original description. Yet the group remains distinct enough for modern descriptions to proceed in want of redescriptions, given the distinctiveness of the dorsal carination patterns across the group.

**Table 1.** Distribution, Marine Ecoregions of the World (MEOW), and size range of world *Austrosyrrhoe* species.

**Scientific name**	**Authority**	**Geographic area**	**MEOW provinces**	**MEOW realms**	**Size (mm)**	**Depth (m)**
*Austrosyrrhoe crassipes*	K.H. Barnard, 1925	Cape Point, South Africa	Agulhas	Temperate southern Africa	4	1280
*Austrosyrrhoe fimbriatus*	(Stebbing & Robertson, 1891)	Firth of Clyde, Irish Sea, Scotland	North European Seas	Temperate Northern Atlantic	unknown	164
*Austrosyrrhoe hamptonae*	sp. nov.	Clarion-Clipperton Zone	Eastern Indo-Pacific	N/A	2.5–5.9	4352– 5109
*Austrosyrrhoe kathleenae*	Lörz & Coleman, 2013	New Zealand	Northern/Southern New Zealand	Temperate Australasia	5	1239
*Austrosyrrhoe rinconis*	J.L. Barnard, 1967	Baja California, USA	Warm Temperate Northeastern Pacific	Tropical Eastern Pacific	3	1205
*Austrosyrrhoe septentrionalis*	Stephensen, 1931	West Greenland, Iceland	Arctic/Northern Temperate Atlantic	Temperate Northern Atlantic	3	2702

In discussing the genus *Austrosyrrhoe*, J.L. Barnard (1972) acknowledged that the genus would benefit from more known species to understand the absence of the molar for placement in the broader family concept. The genus remains consistent for the shape of the gnathopods 1 and 2 propodus. *Austrosyrrhoe* has a relatively small body size (3–5 mm) with fine pereopods which break easily; specimens are most frequently collected as fewer than three individuals in samples. This may lead to the taxon being overlooked in deep sea ecological sampling.

The genus *Syrrhoe* Goës, 1866 includes 15 species; the current material from the Clarion-Clipperton Zone, *S. manowitzae* sp. nov., is the third species described from the north east Pacific, the other two being *S. longifrons* Shoemaker, 1964 and *S. oluta* J.L. Barnard, 1972 (Fig. 8). Pacific specimens are known from 150 to 4209 m depth, including the new species described here. Globally, *Syrrhoe* individuals are known from surface tows to depths of 4209 m. Eleven species are known from less than 800 m depth, with only four species, *S. kareenae* Lörz & Coleman, 2013, *S. oluta*, *S. serrima* J.L. Barnard, 1972, and *S. manowitzae* sp. nov., occurring below 1400 m. As well as an extensive depth range, body size has been recorded as ranging from 3.4 to 14 mm in *Syrrhoe* (Table 2).

**Table 2.** Distribution, Marine Ecoregions of the World (MEOW), and size range of world *Syrrhoe* species.

**Scientific name**	**Authority**	**Geographic area**	**MEOW provinces**	**MEOW realms**	**Size (mm)**	**Depth (m)**
*Syrrhoe affinis*	Chevreux, 1908	Morocco, North Atlantic Coast	Lusitanian	Temperate Northern Atlantic	7	851
*Syrrhoe angulipes*	Ledoyer, 1977	Marseille, France	Lusitanian	Temperate Northern Atlantic	3.5	360
*Syrrhoe anneheleneae*	Fuchs, Coleman & Lörz, 2019	Spitsbergen, Norway	North European Seas	Temperate Northern Atlantic	9	579
*Syrrhoe crenulata*	Goës, 1866	Spitsbergen, Norway	North European Seas	Temperate Northern Atlantic	10	742
*Syrrhoe kareenae*	Lörz & Coleman, 2013	off Oates Land, Ross Sea, Antarctica	Ross Sea, Continental High Antarctic	Southern Ocean	9	1645
*Syrrhoe longifrons*	Shoemaker, 1964	Georges Channel, Vancouver Island, Canada	Cold Temperate North East Pacific	Temperate North Pacific	10	150
*Syrrhoe manowitzae*	sp. nov.	Clarion-Clipperton Zone	Eastern Indo-Pacific	N/A	5.5– 20	4185–4368
*Syrrhoe nodulosa*	K.H. Barnard, 1932	Admiralty Bay, Palmer Archipelago, South Shetland Islands	Scotia Sea	Southern Ocean	14	500
*Syrrhoe oluta*	J.L. Barnard, 1972	Columbia and Oregon, United States	Cold Temperate North East Pacific	Temperate Northern Pacific	7	3251
*Syrrhoe papyracea*	Stebbing, 1888	Culebra Island, West Indies, Caribbean Sea	Tropical Northwestern Atlantic	Tropical Atlantic	13	713
*Syrrhoe petitaserrata*	Hughes, 2009	Great Barrier Reef, Queensland, Australia	Northeast Australian Shelf	Central Indo-Pacific	3.4	0
*Syrrhoe psychrophila*	Monod, 1926	Bellingshausen Sea, Antarctica	Amundsen/Bellingshausen Sea, Continental High Antarctic	Southern Ocean	7.8	400
*Syrrhoe sadiae*	Lörz & Coleman, 2013	Tasman Sea and South Pacific New Zealand	Northern New Zealand	Temperate Australasia	8.5	770
*Syrrhoe semiserrata*	Stebbing, 1888	off Melbourne, Victoria, Australia	Southeastern Australian Shelf	Temperate Australasia	7.6	60
*Syrrhoe serrima*	J.L. Barnard, 1972	off Argentina 40 South	Magellanic	Temperate South America	5.8	1475
*Syrrhoe tuberculata*	Dahl, 1954	Discovery Inlet, Ross Sea	Ross Sea, Continental High Antarctic	Southern Ocean	unknown	550

## Materials and methods

The material for the present study was sampled in the central Eastern Pacific, specifically in the easternmost sector of Clarion-Clipperton Zone (CCZ). The material was collected with epibenthic sledge (EBS) or multi-corer during four scientific deep-sea cruises: the ABYSSLINE-2 (ABYSSal baseLINE project) in 2015, MANGAN 2018, MANGAN 2023, and NORI-D 2021. For details of gear deployment and sample processing, see Jażdżewska and Horton (2026) and Jażdżewska et al. (2025). Samples used in this study were preserved in 80% ethanol. Material is lodged at the Senckenberg Natural History Museum (Frankfurt, Germany; **SMF**) and the Natural History Museum (London, United Kingdom; **NHMUK**). All the remaining material is kept at the Deutsches Zentrum für Marine Biodiversitätsforschung (Wilhelmshaven, Germany; **DZMB**).

Individuals were initially examined using either a Leica M125 or a Nikon SMZ800 stereomicroscope.

Specimens were dissected in 80% ethanol, and microscope slides were made with Polyvinyl Lactophenol or Aquatex mounting agent. Illustrations were made using a Nikon SMZ 800 (whole animal) stereomicroscope and a Nikon Eclipse 50i compound microscope fitted with a camera lucida. The habitus of *Austrosyrrhoe hamptonae* sp. nov. is additionally imaged using a confocal laser scanning microscope (CLSM). The holotype was stained in Congo red and acid fuchsin, temporarily mounted onto slides with glycerine, and examined with a Leica TCS SPV equipped with a Leica DM5000 B upright microscope and three visible-light lasers (DPSS 10 mW 561 nm; HeNe 10 mW 633 nm; and Ar 100 mW 458, 476, 488, and 514 nm), combined with the software LAS AF v. 2.2.1 (Leica Application Suite, Advanced Fluorescence). A series of photographic stacks were obtained, collecting overlapping optical sections throughout the whole preparation (Michels and Büntzow 2010; Kamanli et al. 2017).

Standard abbreviations on the plates are: **A**, antenna; **G**, gnathopod; **H**, head; **LL**, labium; **Md**, mandible; **Mx1**, maxilla 1; **Mx1 P**, maxilla 1 palp; **Mx2**, maxilla 2; **Mxp**, maxilliped; **P**, pereopod; **T**, telson; **U**, uropod; **UL**, labrum; **L**, left; **R**, right. Distributions in Table 1 are by geographic region following Spalding et al. (2007) for Marine Ecoregions of the World (MEOW).

All individuals were subjected to cytochrome *c* oxidase subunit I gene (COI) barcoding prior to identification of the species. The molecular procedures are described in Jażdżewska et al. (2025). All sequences were deposited in GenBank with the accession numbers PQ734342, PQ734362, PQ734459, PQ734493, PQ734518, PQ734598, PQ734626, PQ734672, PQ734768, and PZ022426. The relevant voucher information, taxonomic classifications and sequences are deposited in the data set “DS-AMPHICCZ” in the Barcode of Life Data System (BOLD) (https://doi.org/10.5883/DS-AMPHICCZ) (Ratnasingham and Hebert 2007).

## Systematics

**Family Synopiidae Dana, 1853**

### Genus *Austrosyrrhoe* K.H. Barnard, 1925

#### Austrosyrrhoe hamptonae sp. nov.

Taxon classificationAnimaliaAmphipodaSynopiidae

1F4E481E-1F83-59F8-9421-9465E3326823

https://zoobank.org/D1879238-1B9C-4A43-B838-60A35DDF322A

Figs 1, 2, 3, 8

##### Type material.

***Holotype***: Pacific Ocean • female, 5.9 mm, Clarion-Clipperton Zone; BGR exploration contract area, R/V Sonne, MANGAN 2018, EBS, SO 262-155, 9 May 2018, 11°47.436'N, 117°32.213'W – 11°47.677'N, 117°30.910'W, 4352–4351 m, SMF 63342, COI: PQ734768. ***Paratypes***: Pacific Ocean • juvenile, 2.5 mm, Clarion-Clipperton Zone; BGR exploration contract area, R/V Kilo Moana, MANGAN 2023, EBS, KM23-69, 4 May 2023, 11°36.252'N, 118°02.981'W – 11°37.1050'N, 118°01.2511'W, 4368–4356 m, SMF 63344, COI: PQ734518 • male, size undetermined, Clarion-Clipperton Zone, BGR exploration contract area, R/V Kilo Moana, MANGAN 2023, EBS, KM23-7, 17 Apr 2023, 12°02.2976'N, 138°05.4932'W – 12°03.5888'N, 138°05.7092'W, 5109–5139 m, SMF 63343, COI: PQ734493.

**Figure 1.**
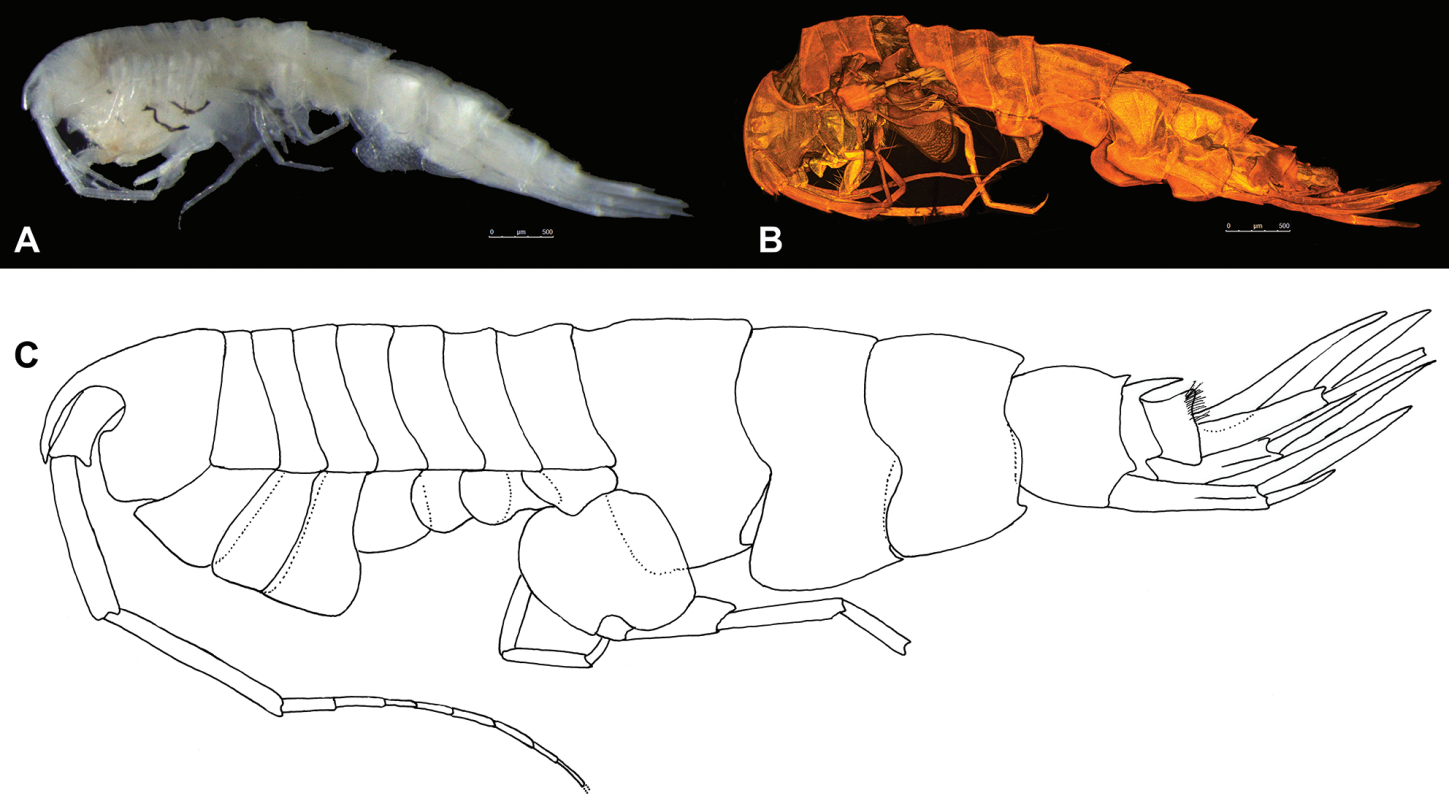
*Austrosyrrhoe hamptonae* sp. nov. **A**. microscope photograph of holotype, female, 5.9 mm, SMF 63342; **B**. CLSM photograph of holotype, female, 5.9 mm, SMF 63342; **C**. habitus of paratype, male, size undetermined, SMF 63343.

**Figure 2.**
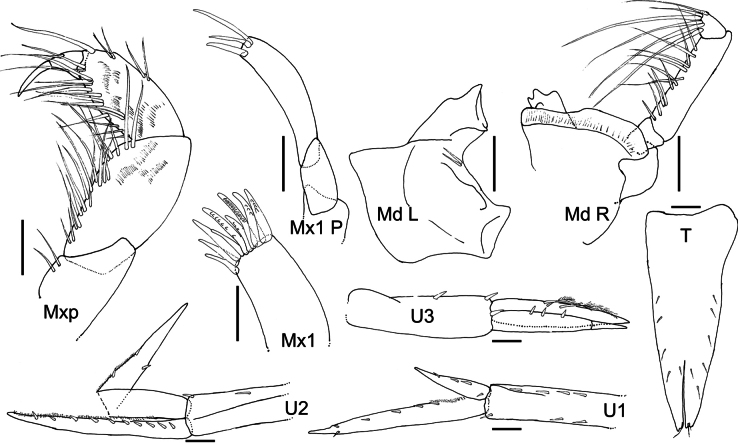
*Austrosyrrhoe hamptonae* sp. nov. Mxp, holotype, female, 5.9 mm, SMF 63342, all other appendages paratype, size undetermined, SMF 63343, Clarion-Clipperton Zone. Scale bar: 0.1 mm.

**Figure 3.**
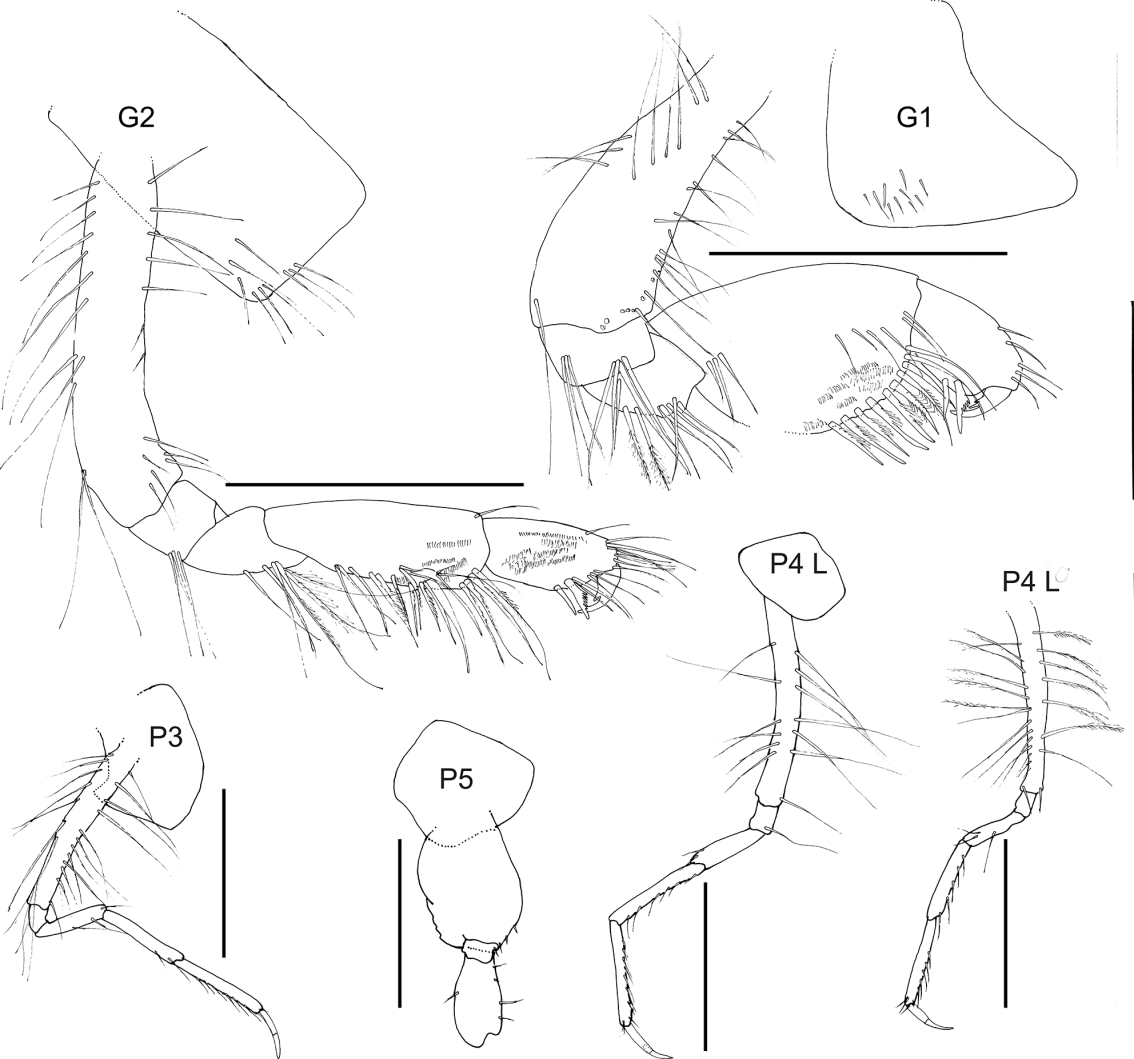
*Austrosyrrhoe hamptonae* sp. nov. G1, G2, P3–5, holotype, female, 5.9 mm, SMF 63342; paratype, size undetermined, P4 L, SMF 63343, Clarion-Clipperton Zone. Scale bar: 0.5 mm.

##### Other material.

Pacific Ocean • unsexed (head only), not measured, Clarion-Clipperton Zone; BGR exploration contract area, R/V Kilo Moana, MANGAN 2023, EBS, KM23-69, 4 May 2023, 11°36.252'N, 118°02.981'W – 11°37.1050'N, 118°01.2511'W, 4368–4356 m, DSB_8459, COI: PQ734598.

##### Diagnosis.

Gnathopods 1–2 propodus palm defined by serrate robust seta. Pereopod 3 coxa anterodistal lobe produced subovate, posterodistal lobe absent. Pereopods 5–6 basis subovoid. Pleonites 1–3 posterodistal margin smooth with single acute convex dorsal margin, with posterior minute dorsal process. Epimera 1–3 posterior margin concave, posterodistal corner with small acute tooth. Urosomite 1 dorsal margin smooth with small acute dorsal process. Urosomite 2 dorsal margin smooth with acute process directed posteriorly. Urosomite 3 posterior margin with setal fringe.

##### Description.

***Head*** (Figs 1, 2) not protuberant; rostrum long, directed ventrally, apically acute; lateral cephalic lobe subtriangular; eyes unknown; accessory eyes unknown. Antenna 1 unknown. Antenna 2 flagellum more than 7-articulate, broken. Mandible palp article 2 with many slender setae; article 3 with many slender apical setae; right lacinia mobilis 3 dentate; molar greatly enlarged, smooth, not triturative, accessory setal row with two setae on left. Maxilla 1 inner plate unknown, outer plate with nine robust setae, palp article 2 with three apical robust setae. Maxilliped not foliaceous.

***Pereon*** (Figs 1, 3). Pereonites 1–7 dorsal margin smooth, without lateral ridge. Gnathopod 1 subchelate; coxa anterior margin produced, rounded, not tapering distally; basis length subequal to carpus; propodus palm subacute/oblique, defined by serrate robust seta; dactylus well developed, with posterior margin with several teeth. Gnathopod 2 subchelate; coxa not distally tapering; basis longer than carpus; propodus palm subacute/oblique, defined by one serrate and one smooth robust seta; dactylus well developed, posterior margin with one tooth. Pereopod 3 pelagont (dominant over coxa 4), coxa anterodistal lobe produced subovate, posterodistal lobe absent. Pereopod 4 coxa smaller than coxa 3, lobate in shape, with well-developed posteroventral lobe half the depth of the posterior margin. Pereopods 5 and 6 bases subovoid, posterodistal lobe not extending below ischium, posterior margin broadly serrate. Pereopod 5 basis posterior margin weakly subquadrate.

***Pleon*** (Figs 1, 2). Pleonites 1–3 posterodistal margin smooth with single acute, convex dorsal margin, with posterior minute dorsal process. Epimera 1–3 posterior margin concave, posterodistal corner with small acute tooth. Urosomite 1 dorsal margin smooth with small acute dorsal process. Urosomite 2 dorsal margin smooth with acute process directed posteriorly. Urosomite 3 posterior margin with setal fringe. Uropod 3 length exceeding uropods 1 and 2; peduncle long, length twice breadth. Telson length more than two times breadth (broken), longer than uropod 3 peduncle, moderately cleft along 1/3 length, without setae along the lateral margin, apical setae unknown.

##### Etymology.

Named for Jacqueline B. Hampton, who illustrated amphipods of the north eastern Pacific from the Cedros Trench and offshore of the Columbia River, Oregon, in the 1960s and 1970s publications of Jerry Barnard.

##### Molecular identification.

Following the definition given by Pleijel et al. (2008), the sequence of the holotype male of *Austrosyrrhoe hamptonae* sp. nov. (SMF 63342, GenBank accession number PQ734768) is designated as a hologenophore of all obtained sequences. The sequences of the paratypes and non-type collection of the species are deposited in GenBank with the following accession numbers: PQ734493, PQ734518, PQ734598. The species has also received a Barcode Index Number from Barcode of Life Data Systems: BOLD:AEB6000 (https://doi.org/10.5883/BOLD:AEB6000).

##### Remarks.

*Austrosyrrhoe hamptonae* sp. nov. can be separated from other species in the genus by the urosomite dorsal carination pattern, with urosomite 1 dorsal margin smooth with a small acute tooth, urosomite 2 dorsal margin with an acute process, and urosomite 3 posterior margin with a setal fringe.

*Austrosyrrhoe hamptonae* sp. nov. and *A. fimbriatus* (Stebbing & Robertson, 1891) both have the urosomite 3 posterodorsal margin lined with a setal fringe. *Austrosyrrhoe hamptonae* sp. nov. can be separated from *A. fimbriatus* and *A. septentrionalis* Stephensen, 1931 by the pereopods 5 and 6 bases being subovate in *A. hamptonae* sp. nov. and more subquadrate-rectilinear in the latter species.

*Austrosyrrhoe rinconis* J.L. Barnard, 1967 is reported from nearby Baja California (1095–1205 m), while the remaining four species are in the North Atlantic, South Atlantic, and South Pacific Ocean (Fig. 7). *Austrosyrrhoe hamptonae* sp. nov. has the coxa 3 anterior distal corner rounded while in *A. rinconis* the corner is acute.

*Austrosyrrhoe hamptonae* sp. nov., *A. crassipes* K.H. Barnard, 1925, and *A. kathleenae* Lörz & Coleman, 2013 have a single serrate palm defining setae on gnathopods 1 and 2, while *A. septentrionalis* has gnathopods 1 and 2 with two setae defining the palm and in *A. fimbriatus* the palm-defining setae are simple.

A search of the NHM collection for the comparative type material of *A. fimbriatus* concluded the material is lost. Previous work by J.L. Barnard (1972) stated the type specimens of *A. crassipes* were in poor condition and beyond repair to properly diagnose the genus.

Prior to the current study, the deepest record of *Austrosyrrhoe* was *A. septentrionalis* at 2702 m near Greenland. The material of *A. hamptonae* from depths of 4352–5109 m in the Clarion-Clipperton Zone, Pacific Ocean increases this by more than 2409 meters.

### Genus *Syrrhoe* Göes, 1866

#### Syrrhoe manowitzae sp. nov.

Taxon classificationAnimaliaAmphipodaSynopiidae

D09A76EA-781D-55A5-8680-10C5F1BA7238

https://zoobank.org/6AFAC766-4CC3-4D9F-8954-8527696D661C

Figs 4, 5, 6, 7, 8

##### Type material.

***Holotype***: Pacific Ocean • 1 female, 20 mm; Clarion-Clipperton Zone; 11.604°N, 118.05°W; depth 4368 m; 31 May 2021; NORI-D exploration contract area, NORI-D 2 cruise MC_140, RV Maersk Launcher; multi-corer; NHMUK 2025.2873, COI (PZ022426). ***Paratypes***: Pacific Ocean • female, 19 mm; Clarion-Clipperton Zone; UKSR-1 exploration contract area, R/V Thompson, ABYSSLINE-2, EBS, AB2-EB01, 18 Feb. 2015, 12°22.02'N, 116°33.00'W, 4352 m, SMF 63361, COI: PQ734459 • juvenile, 8 mm; Clarion-Clipperton Zone; UKSR-1 exploration contract area, R/V Thompson, ABYSSLINE-2, EBS, AB2-EB07, 02 Mar. 2015, 12°27.06'N, 116°37.80'W, 4145 m, SMF 63362, COI: PQ734626 • juvenile, 6 mm; Clarion-Clipperton Zone; BGR exploration contract area, R/V Kilo Moana, EBS, KM23-50, 1 May 2023, 11°17.7919'N, 116°18.8626'W – 11°18.5445'N, 116°17.6747'W, 4185–4182 m, SMF 63364, COI: PQ734672 • juvenile, 5.5 mm; Clarion-Clipperton Zone; BGR exploration contract area, R/V Kilo Moana, EBS, KM23-50, 1 May 2023, 11°17.7919'N, 116°18.8626'W – 11°18.5445'N, 116°17.6747'W, 4185–4182 m, SMF 63363 COI: PQ734362

**Figure 4.**
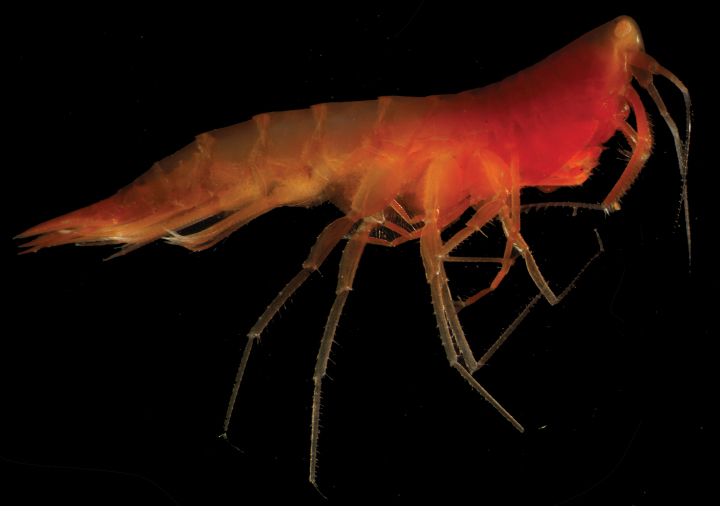
*Syrrhoe manowitzae* sp. nov., microscope photograph, female, 20 mm, NHMUK 2025.2873, Clarion-Clipperton Zone.

**Figure 5.**
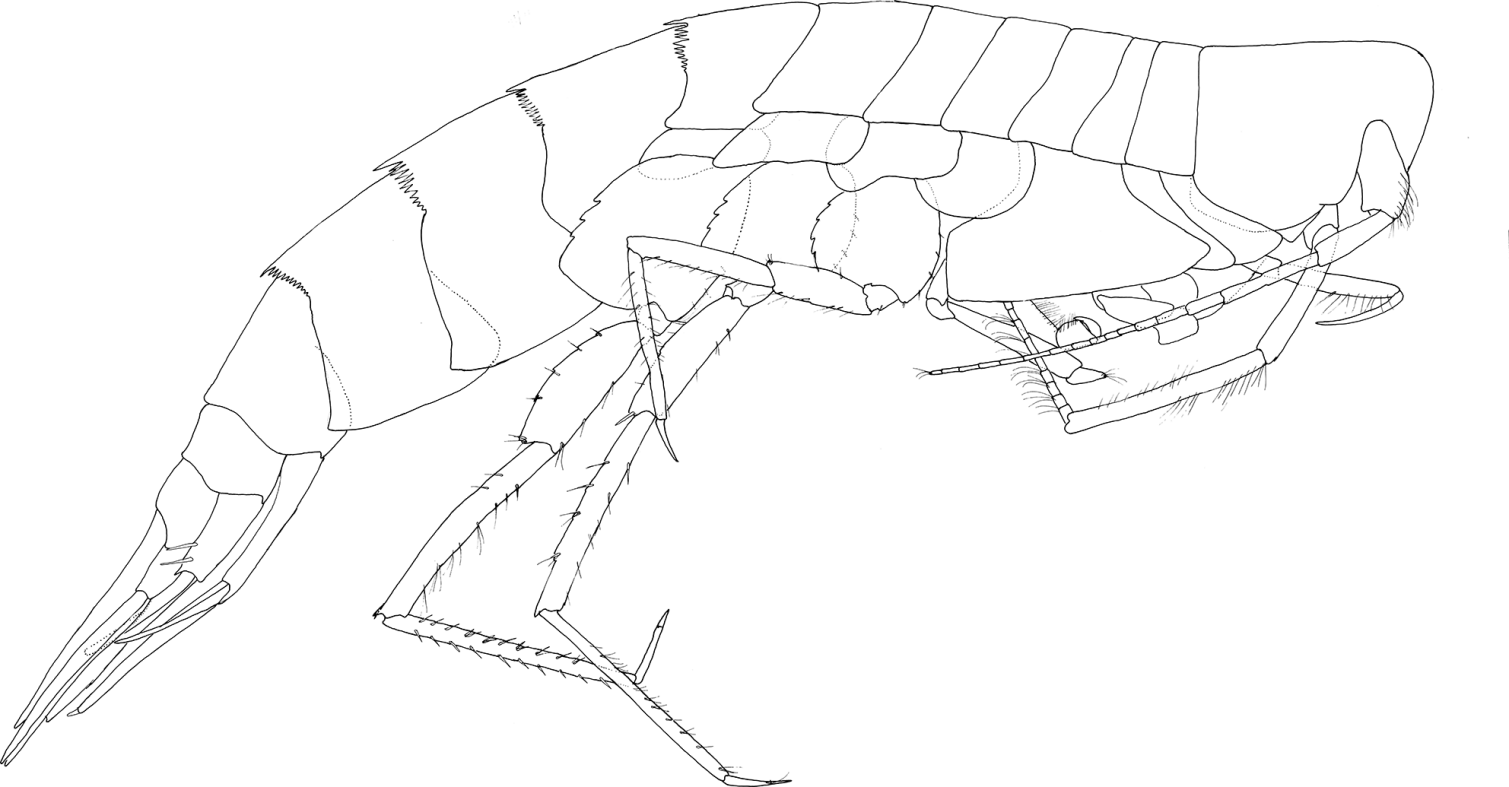
*Syrrhoe manowitzae* sp. nov., holotype, female, 20 mm, NHMUK 2025.2873, Clarion-Clipperton Zone.

**Figure 6.**
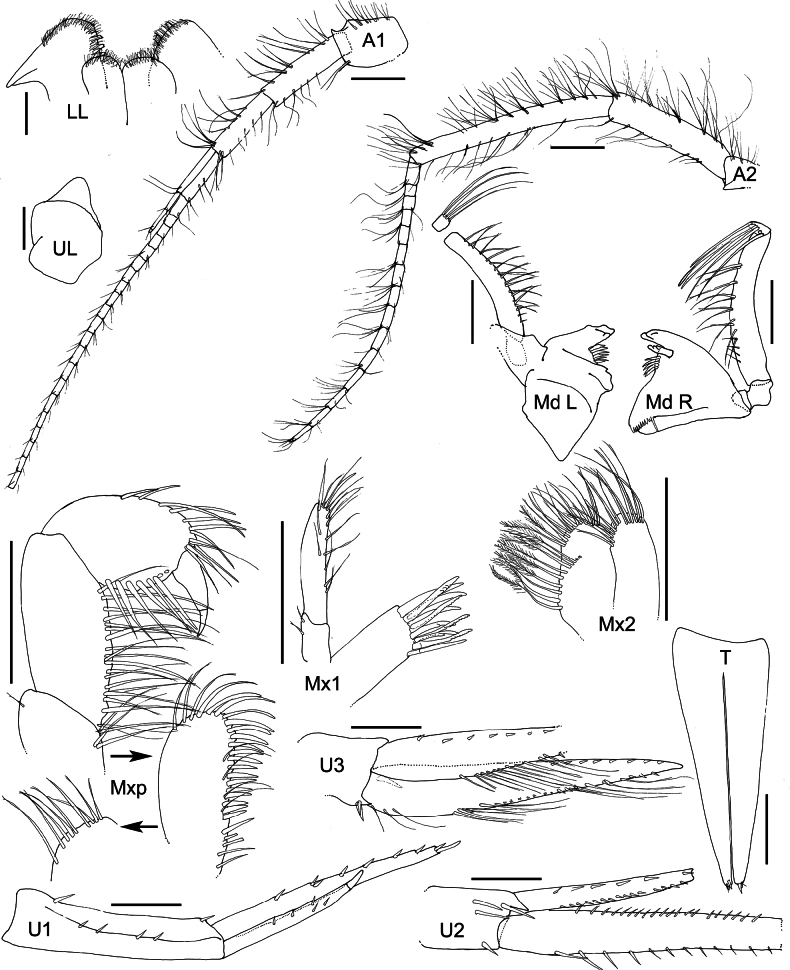
*Syrrhoe manowitzae* sp. nov., holotype, female, 20 mm, NHMUK 2025.2873, Scale bar: 0.5 mm.

**Figure 7.**
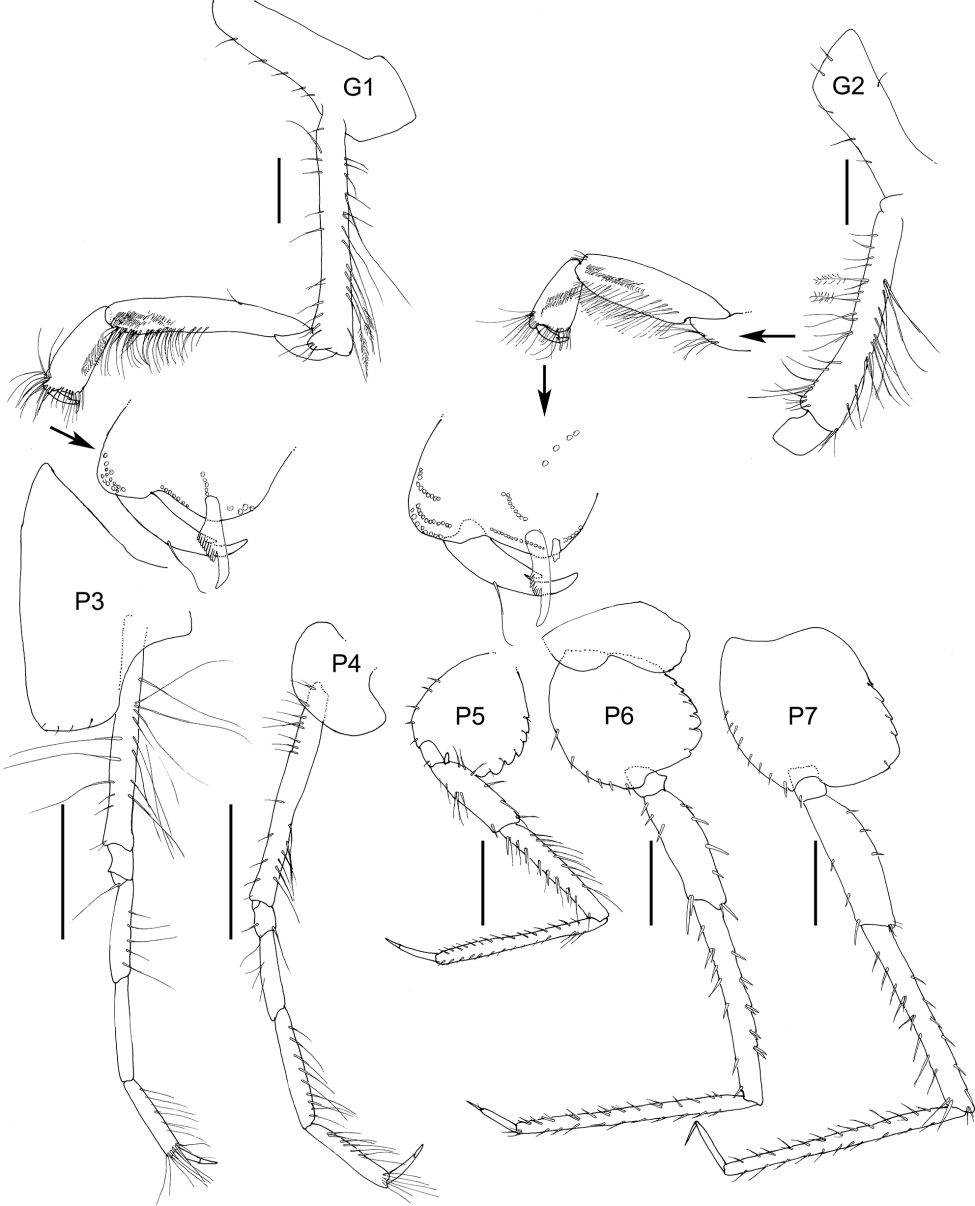
*Syrrhoe manowitzae* sp. nov., holotype, female, 20 mm, NHMUK 2025.2873. Scale bar: 1 mm.

**Figure 8.**
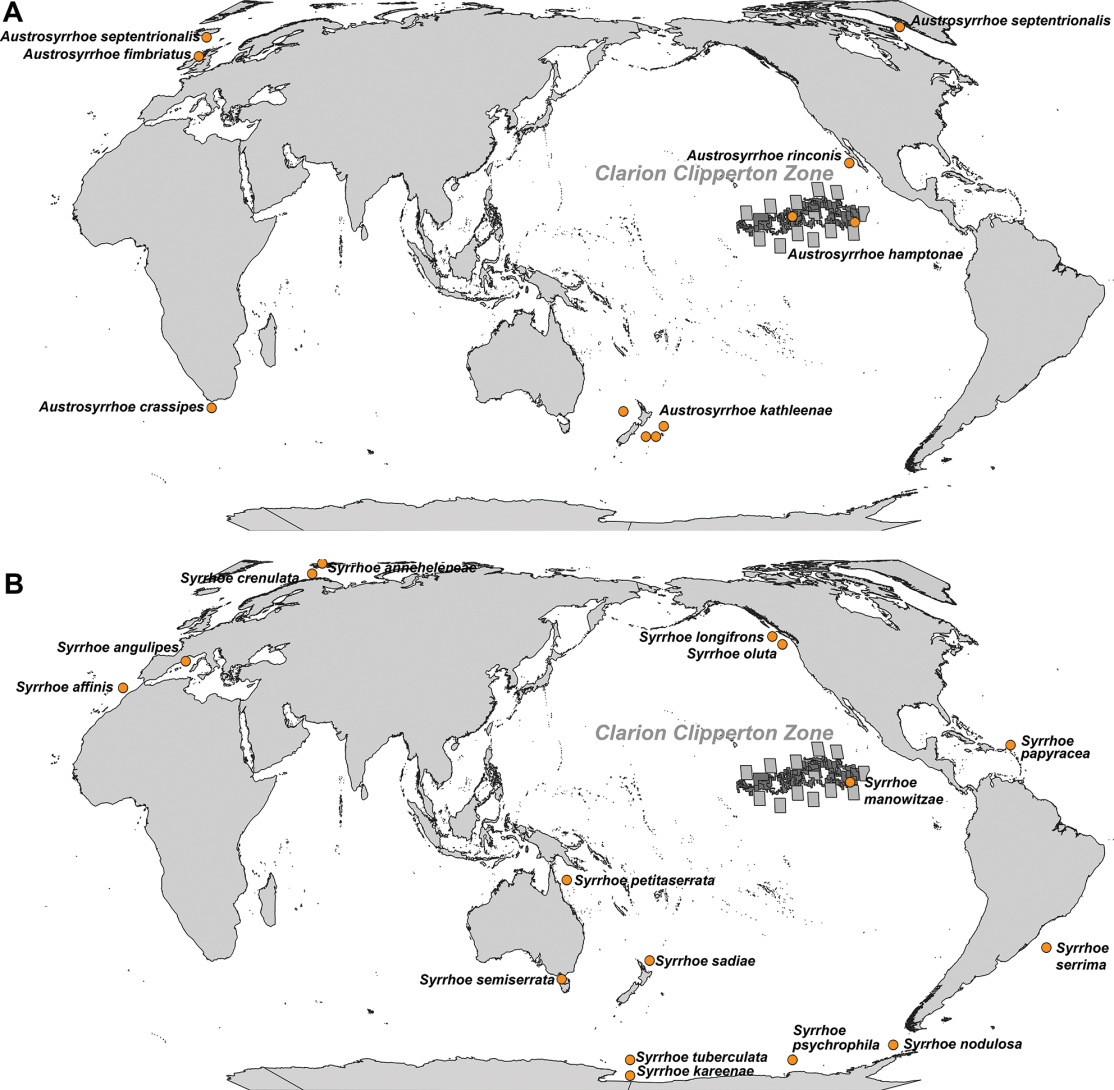
Distribution map of world. **A**. *Austrosyrrhoe*; **B**. *Syrrhoe*.

##### Other material.

Pacific Ocean • juvenile, 6 mm; Clarion-Clipperton Zone; BGR exploration contract area, R/V Kilo Moana, EBS, KM23-69, 4 May 2023, 11°36.252'N, 118°02.981'W – 11°37.1050'N, 118°01.2511'W, 4368–4356 m, DSB_8423), COI: PQ734342

##### Diagnosis.

Head protuberant. Pereonite 7 posterodistal margin minutely serrate with single acute dorsal tooth. Epimera 1–3 posterior margin smooth, posterodistal corner subquadrate. Telson length 2.5 times breadth, longer than uropod 3 peduncle, deeply cleft (90% of telson depth), without setae along the lateral margin, with single pair of short robust apical setae.

##### Description.

Colour in life bright red, transitioning to lighter orange posteriorly.

***Head*** (Figs 4, 5, 6) protuberant; rostrum short, directed ventrally, apically acute; lateral cephalic lobe absent; eyes pale pink/orange ommatidia in white matrix (not apparent in preserved material); accessory eyes absent. Antenna 1 peduncular article 1 not elongate, with an anterior distal tooth; peduncular article 2 not elongate; flagellum shorter than pereon. Antenna 2 flagellum 16-articulate. Mandible palp article 2 with many slender setae; article 3 with many slender apical setae; lacinia mobilis tri-dentate; molar greatly enlarged, smooth, not triturative; right accessory setal row with five serrate setae; left accessory setal row with seven serrate setae. Maxilla 1 inner plate unknown, outer plate with ten robust setae, palp article 2 lined with medial and apical slender setae. Maxilliped not foliaceous.

***Pereon*** (Figs 4, 5, 7). Pereonites 1–6 dorsal margin smooth, without lateral ridge. Pereonite 7 posterodistal margin minutely serrate with single acute dorsal tooth. Gnathopod 1 subchelate; coxa anterior margin not produced, not tapering distally; basis longer than carpus; propodus palm nearly transverse, defined by a serrate robust seta. Gnathopod 2 subchelate; coxa not distally tapering; basis longer than carpus; propodus palm nearly transverse, defined by a serrate robust seta; dactylus well developed. Pereopod 3 coxa anterodistal lobe produced and subacute, posterodistal lobe margin smooth, truncate, depth 1/2 of coxa. Pereopod 4 coxa smaller than coxa 3, lobate in shape, with weakly developed posteroventral lobe. Pereopods 5–7 basis subovoid, expanded, posterodistal lobe not extending below ischium, anterodistal corner rounded, posterior margin broadly serrate.

***Pleon*** (Figs 5, 6). Pleonites 1–3 posterodistal margin minutely serrate, with single acute dorsal tooth, without lateral ridges. Epimera 1–3 posterior margin smooth, posterodistal corner subquadrate. Uropod 3 length exceeding uropods 1 and 2; peduncle short, length twice breadth. Telson length 2.5 times breadth, longer than uropod 3 peduncle, deeply cleft (90% of telson depth), without setae along the lateral margin, with single pair of short robust apical setae.

##### Etymology.

Named for Naomi Manowitz, who illustrated amphipods from the north eastern Pacific offshore of the Columbia River, Oregon, in the 1970s publications of Jerry Barnard.

##### Molecular identification.

Following the definition given by Pleijel et al. (2008), the sequence of the holotype male of *S. manowitzae* sp. nov. (NHMUK 2025.2873, GenBank accession number PZ022426) is designated as a hologenophore of all obtained sequences. The sequences of the paratypes and non-type collection of the species are deposited in GenBank with the following accession numbers: PQ734342, PQ734362, PQ734459, PQ734626, PQ734672. The species has also received a Barcode Index Number from Barcode of Life Data Systems: BOLD:AEA8314 (https://doi.org/10.5883/BOLD:AEA8314).

##### Remarks.

The protuberant head of *Syrrhoe manowitzae* sp. nov. aligns it with three other *Syrrhoe* species, namely *S. oluta* J.L. Barnard, 1972, *S. papyracea* Stebbing, 1888, and *S. longifrons* Shoemaker, 1964. *Syrrhoe manowitzae* sp. nov. is most closely related to *S. oluta* and *S. longifrons* based on the long, deeply cleft telson. *Syrrhoe manowitzae* sp. nov. has a tooth flanked by serrations on pleonite 7 and pleonites 1 and 2, which separates it from *S. oluta* and *S. longifrons* that have smooth dorsal margins across all posterior somites (pereonite 7, pleonites 1 and 2). Eyes sizes also differ with *S. manowitzae* sp. nov. having much smaller compound eyes in comparison to *S. longifrons* in where the eyes are very large (Fig. 4).

The single dorsal tooth with minute lateral serrations (pereonite 7, pleonites 1 and 2) on *S. manowitzae* sp. nov. is similar to *S. serrima* J.L. Barnard, 1972 (5.8 mm, Argentina); these species differ in the epimera 3 posterior margin which is smooth in *S. manowitzae* sp. nov. and serrate in *S. serrima*.

*Syrrhoe manowitzae* sp. nov. individuals were recorded from similar depths despite the significant difference in size of individuals: 4185–4368 m depth and 5.5–20 mm in body size.

## Discussion

There are just two sequences identified as *Austrosyrrhoe* in publicly available databases. These two sequences of a single MOTU (BOLD:ADG8411) are from the North Atlantic (Jażdżewska et al. 2018) and show a 20% *p*-distance dissimilarity to the sequences of *A. hamptonae* sp. nov.

There are eight sequences identified as *Syrrhoe* in publicly available databases. These sequences represent six MOTUs (BOLD:AAG7107, BOLD:AAG7108, BOLD:ABY3672, BOLD:ACE3877, BOLD:ACM1995, and BOLD:ADH2660) from the North Atlantic (Jażdżewska et al. 2018; boldsystems.org) and have a ~28% *p*-distance dissimilarity to the sequences of *S. manowitzae* sp. nov.

The two new Clarion-Clipperton Zone synopiid species described here are known only from ten specimens in total. The four records of *A. hamptonae* sp. nov. between 4352 and 5109 m significantly increase the known depth range of the genus previously known from ~2700 m. The six specimens of *S. manowitzae* sp. nov. are from a similar depth range of 4185–4368 m whereas the previous deepest record for the genus was 3251 m.

## Supplementary Material

XML Treatment for Austrosyrrhoe hamptonae

XML Treatment for Syrrhoe manowitzae

## Additional information

### Use of AI

No use of AI was reported.

### Author ORCIDs

Lauren Elizabeth Hughes https://orcid.org/0000-0002-5679-1732

Anne Helene Solberg Tandberg https://orcid.org/0000-0003-3470-587X

### Data availability

All of the data that support the findings of this study are available in the main text.
